# Investigation of the Dynamic Stiffness of Poroelastic and Asphalt Concrete Layers under In Situ and Laboratory Conditions

**DOI:** 10.3390/ma15051821

**Published:** 2022-02-28

**Authors:** Krzysztof Robert Czech, Wladyslaw Gardziejczyk

**Affiliations:** 1 Department of Geotechnics and Structural Mechanics, Faculty of Civil Engineering and Environmental Sciences, Bialystok University of Technology, Wiejska 45E, 15-351 Bialystok, Poland; 2 Department of Construction and Road Engineering, Faculty of Civil Engineering and Environmental Sciences, Bialystok University of Technology, Wiejska 45E, 15-351 Bialystok, Poland; w.gardziejczyk@pb.edu.pl

**Keywords:** dynamic stiffness, wearing course, poroelastic pavements, test stand

## Abstract

Compounds with lower dynamic stiffness are a better solution from the tyre/road noise point of view. The article presents the constructed test stand for the evaluation of dynamic stiffness both in in situ and in laboratory conditions. As a result of the tests, it was found that poroelastic pavements have a much lower dynamic stiffness (from 138.3 to 143.0 dB re. 1 N/m) compared to the asphalt concrete pavement (150.3 dB re. 1 N/m). In the group of poroelastic pavements, lower dynamic stiffness is characteristic for pavements with a binder course of porous asphalt. The results of the research are a contribution to further work on the influence of the dynamic stiffness of the pavements on the tyre/road noise level. The conducted measurements and analysis of the results prove the usefulness of the proposed test stand for determining the dynamic stiffness of bituminous mixtures in laboratory and field conditions. This is confirmed by the coherence between the force and acceleration signals at the level of at least 0.96—which indicates a very good validation of the test results with a random error lower than ±5% with 90% confidence level.

## 1. Introduction

Solutions with a poroelastic wearing course are a special type of road pavement. Such a surface is characterized by the fact that the upper layer of the surface is made of a mineral-rubber-asphalt mixture. Such a mixture contains more than 20% air void content and about 20% rubber granules. A poroelastic pavement has a positive effect on the reduction in the tyre/road noise level. This is due to better sound absorption because of high void content, and lower stiffness as a result of the use of rubber granulate to partially replace mineral aggregate. Research on poroelastic pavement was carried out as part of the SEPOR project (TECHMATSTRATEG1/347040/17/NCBR/2018) [1]. A mineral-rubber-asphalt mix with a maximum aggregate grain size of 8 mm (designated as PSMA8) and a maximum aggregate grain size of 5 mm (designated as PSMA5) was embedded in the wearing course of poroelastic pavement. The binder course was a mix of stone mastic asphalt (SMA 11) and porous asphalt (PA11).

**Figure 1:**
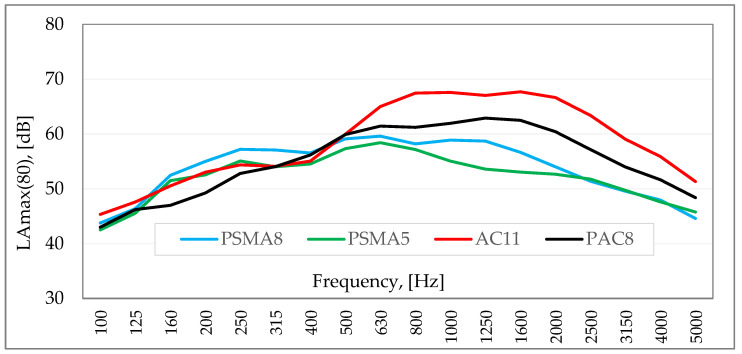
Sound spectra of a statistical passing of a passenger vehicle travelling at a speed of 80 km/h on four surfaces (PSMA8, PSMA5, AC11, PAC8).

Figure 1 shows the sound spectra of a statistical passenger vehicle travelling at a speed of 80 km/h on four surfaces (PSMA8—poroelastic pavement with a maximum grain size of 8 mm, PSMA5—poroelastic pavement with a maximum grain size of 5 mm, AC11—asphalt concrete with a maximum aggregate grain size of 11 mm, PAC8—porous asphalt with a maximum aggregate grain size of 8 mm), determined according to the method of controlling travelling. The PSMA8, PSMA5 and AC11 pavements were built as test sections under the SEPOR project, and the PAC8 pavement was used on one of the provincial roads in Poland.

Differences in sound levels in the frequency range above 500 Hz are partly due to the different grain size of the aggregate and different void content. Considering the same maximum aggregate grain size of PSMA8 and PAC8 mixtures and similar void content, it can be assumed that the differences in sound levels result from varying levels of stiffness of these layers. Contrary to the assessment of the load capacity of road surfaces, in the case of tyre/road noise, pavements with lower stiffness are the better solution. In the literature, despite general records on the impact of the stiffness of the upper pavement layers on the tyre/road noise level, there are no clear findings in this regard.

Results of testing of the rolling noise of car tyres confirm the need to develop a test stand that could be used to reliably determine both the dynamic stiffness of bituminous mixtures in laboratory conditions and road pavements in field conditions.

The first configuration of the test stand was presented in [2]. This paper presents the modifications introduced in the previously built test stand and also presents the results of dynamic stiffness measurements carried out directly on poroelastic pavements and on asphalt concrete pavement (in situ tests), as well as the results of tests carried out in laboratory conditions on samples cut from these pavements.

## 2. Literature Review

The dynamic stiffness of asphalt mixtures is usually determined under laboratory conditions. Such tests can be carried out with the use of universal testing machines or other dedicated machines and devices. Usually, they are also equipped with thermal chambers that enable the testing of samples under various temperature conditions.

The detailed method of preparing bituminous samples and performing stiffness measurements is presented in standard DIN EN 12697-26:2012 [3]. In accordance with the above-mentioned standard, stiffness can be determined in:

- a two-point bending test on trapezoidal specimens or prismatic specimens (2PB-TR and 2PB-PR tests, respectively),
- a three-point bending test on prismatic specimens or a four-point bending test on prismatic specimens (3PB-PR and 4PB-PR tests),
- an indirect tensile test on cylindrical specimens (with the use of devices for applying repeated load pulse indirect tension—IT-CY tests, or a cyclic indirect tension—CIT-CY test);
- a direct uniaxial test, in which cylindrical specimens are subjected to direct tension-compression (DTC-CY test) or only direct tension (DT-CY test). In the latter case, when the test consists only of direct tension, the specimen may also have a prismatic shape (DT-PR test).

The research methods specified in standard [3] were used, inter alia, in tests of asphalt specimens presented in the works [4,5,6]. Mackiewicz and Szydło [4] for determining the complex stiffness modulus E* and phase angle φ used the four-point bending test on prismatic specimens (4PB-PR). The tests were carried out for the frequency of 10 Hz and four temperatures (−5 °C, 0 °C, +10 °C, and +25 °C). Mazurek and Iwański [5] investigated the dynamic stiffness modulus using the direct tension-compression test (DTC-CY). The tests were carried out for six different frequencies (0.1 Hz, 0.3 Hz, 1 Hz, 3 Hz, 10 Hz and 20 Hz) and five temperatures (−7 °C, +5 °C, +13 °C, +25 °C and +40 °C). In the studies of Li et al. [6] for determining the resilient modulus of the thin layer surfacing cores the repeated load pulse indirect tension test (IT-CY) was used. The tests were carried out for five different temperatures (+5 °C, +10 °C, +15 °C, +20 °C, +25 °C).

A slightly different methodology for determining the dynamic modulus for bitumen samples in laboratory conditions was proposed in the report of the American Transportation Research Board [7] and standard [8], in which the sample is subjected to cyclic uniaxial sinusoidal compression (and not cyclic direct tension (DT-CY test) or direct tension-compression (DTC-CY test)—as was the case in the tests carried out in accordance with standard [3]). This is how the dynamic modulus was determined in [9,10]. In [9], a dedicated “Dynamic Shear Rheometer” (DSR) device was used for this purpose, which enables setting loads in the frequency range from 0.01 Hz to 25 Hz. The tests were carried out for three different temperatures: +4 °C, +20 °C and +40 °C. However, the paper does not indicate the specific frequencies for which the measurements were carried out. In [10], the dynamic modulus was tested with the use of the servo-hydraulic testing system MTS 370.10. The tests were carried out for five temperatures (−10 °C, +4 °C, +21 °C, +37 °C, and +54 °C) and six frequencies (0.1 Hz, 0.5 Hz, 1 Hz, 5 Hz, 10 Hz and 25 Hz).

In a very similar way, when using the repetitive sinusoidal dynamic compressive-axial load, the dynamic modulus of bituminous samples under laboratory conditions was also tested by Walubita et al. [11] and Zhang et al. [12] (in accordance with the guidelines of the older standard [13]). In the first case [11], the tests were carried out for 10 different bituminous mixtures, 3 frequencies (0.1 Hz, 1 Hz and 10 Hz) and 5 different temperatures (−10 °C, +4.4 °C, +21.1 °C, +37.8 °C and +54.4 °C). In the second case [12], the dynamic modulus was tested for six different types of bituminous mixtures at three frequencies (0.1 Hz, 5 Hz and 10 Hz) and two temperatures (+37.8 °C and +54.4 °C).

In laboratory conditions, attempts were also made to test the dynamic stiffness of samples from bituminous mixtures in the same way as was developed for materials used under floating floors in dwellings. The detailed method of specimen preparation and the procedure for testing the dynamic stiffness of these types of material are included in standard EN 29052-1 [14]. The assumptions of this standard were used in study [15] to determine the dynamic stiffness of two samples of Stone Mastic Asphalt (SMA) mix with significantly different densities and samples of other materials (expanded polystyrene (EPS), polyurethane (PU) and concrete). The main difference in the test procedure proposed by Vázquez and Paje (called the Resonant Method) was that the tests were carried out on cylindrical samples with surfaces aligned with a thin layer of plaster of Paris and placed directly on the laboratory floor—while according to standard [14], the sample should be rectangular and set on a base plate. Similarly to the tested sample, the load plate used in tests [15] had the shape of a steel cylinder—with a height selected so that its mass corresponded to 2 kPa of the static load exerted on the tested sample. In order to determine the extrapolated vertical resonance frequency of the sample with the loading mass pressing it, excitation was induced with a sinusoidal sweeping signal, which was carried out by means of a small modal exciter attached to a three-legged tripod in the frequency range from 10 Hz to 1000 Hz. A number of years later, the authors of paper [16] conducted analogous tests of dynamic stiffness with the use of the Resonant Method in the frequency range from 10 Hz to 7 kHz. As before, two types of SMA bituminous mixtures with significantly different stiffness modulus were tested. Measurements of the dynamic stiffness for both bituminous mixtures were carried out for three samples taken during the construction of the road.

The stiffness measurements carried out in laboratory conditions are closely related to the geometry and dimensions of the tested samples and do not take into account the influence of the stiffness of the lower structural layers of the pavement on the tested stiffness. The method of preparing/levelling the surface of the samples (or the lack of their preparation) may also significantly affect the obtained results. Therefore, more and more attempts are being made to test dynamic stiffness directly on road surfaces.

Field research is most often based on impulse excitation realized with impact/modal hammers and the recording of motion response with one or more accelerometers (or other types of transducers) bound or bolted to the wearing course of the pavement. An example of this type of research is described in studies [17,18]. To determine the transfer function and mechanical impedance [Ns/m], an impact hammer was used with an impedance head (direct point) and an accelerometer (transfer point) bound to the road surface at a distance of 10 cm from one another. The tests were carried out on the upper surface of five different poroelastic road surfaces (PERS). In each of the test sites, a 6-fold impulse excitation was performed (an impact hammer hit against a circular steel plate mounted on the upper surface of the impedance head). A similar test procedure was used in laboratory conditions on cuboidal samples bound with polyurethane binder to a massive concrete base with dimensions of 1 m × 1 m.

Pulse excitation to test the stiffness of asphalt pavements was also used in the experimental device “The PiScan Probe” [19]. In addition to the impact hammer, the device had two accelerometers connected with a special frame and weighted from the top with additional mass to ensure better contact of the transducers with the road surface. The distance between the accelerometers could be regulated—15 cm or 30 cm (it was 30 cm during the tests). The pavement stiffness expressed by the average elastic modulus and, additionally, its thickness were determined using the method of Enhanced Resonance Search (ERS) in 15 different testing sites. The algorithm of the ERS method—which is a combination of the SASW (Spectral Analysis of Surface Waves) and Resonant Method (previously used for the same purpose in the case of concrete pavements), is described in detail in [20].

Unfortunately, despite the fact that the impact excitation generated with the use of modal hammers are quick, uncomplicated and convenient in the implementation of various types of field measurements (mainly due to the fact that they do not require any special fixture to the tested structure), they do not provide such a high Signal-to-Noise ratio as the excitations generated by the use of modal shakers/exciters [21]. Impact excitations generated with modal hammers are a very good solution for testing smaller, homogeneous structures. On the other hand, excitations generated with the use of modal shakers/exciters (e.g., random noise, sine sweeps, etc.) prove to be much more effective with more complex structures—including non-linear structures [21,22].

Most probably, it is the significant non-linearity (typical of asphalt mixtures) and the use of impulse excitation to test the stiffness of the road surface that should explain such large differences between the determined (3.9–17.7 GPa) and the expected (2.5–3.2 GPa according to the IKRAM Group Sdn. Bhd. Standard) values of elastic modulus and a difference of up to 56% between what was expected and what was determined with the use of the “The PiScan Probe” to assess the thickness of the asphalt layer [19].

That is why a reliable determination of asphalt pavement stiffness in in situ conditions should be based on modal exciters rather than on impulse excitation.

A good starting point for developing a methodology for this type of research could be laboratory methods using portable modal exciters to determine various types of measures of stiffness of the tested material. Unfortunately, the stiffness of the asphalt pavement cannot be determined in situ in accordance with the guidelines of standard [14]. The procedure for determining the apparent dynamic stiffness per unit area can be used only in the case of samples with specific dimensions and masses. Moreover, asphalt pavements are subject to much more intense and complex loads [23] than the materials used for vibration isolation of floating floors, to which the standard applies [14].

A much better solution when it comes to the possibility of conducting field measurements aimed at reliable determination of the dynamic parameters of asphalt mixtures, equated with their stiffness, is the application of the guidelines used in modal analysis and dynamic diagnostics of buildings, machines, and various types of vehicles, which are included in ISO 7626-2:1990 [24]. The standard indicates the appropriate method of determining the frequency response function, which is mechanical mobility [m/Ns]), using single-point translational excitation with an attached vibration exciter. According to the information contained in [24], in order to determine the mobility function (or any other frequency response function) it is possible to use any excitation waveform (discretely stepped or slowly swept sinusoidal excitation, stationary random excitation, pseudo-random/periodic-chirp/periodic-impulse or periodic-random excitation) generated with the use of electrohydraulic, electrodynamic or piezoelectric exciters—provided that the frequency spectrum covers the frequency of interest.

The solution to enable testing the driving-point dynamic stiffness of asphalt mixtures at the field site in a manner similar to that described in [24] was presented by Vázquez and Paje in [15]. The Non-resonant Method (or the impedance method) does not require the application of a loading mass on the entire upper surface of the sample, or determination of the resonant frequencies of the sample-mass system—as was the case with the determination of the apparent dynamic stiffness per unit area of the test specimen in accordance with the guidelines of standard [14]. In the case of the Non-resonant Method proposed in [15], a small vibration exciter (suspended from a tripod with a pinion-crank system), a stinger and an impedance head attached to a circular plate with a diameter of 14 mm were used to test the dynamic stiffness of various types of construction materials (including samples from SMA bituminous mixtures). In the study, random excitation was used. It was shown in [15] that the dynamic stiffness of asphalt mixtures can be determined using the Non-resonant Method, provided that all tests be carried out on samples of the same shape and dimension. The main advantage of the Non-resonant Method is that it can be carried out in situ. Unfortunately, Vázquez and Paje only carried out laboratory tests on samples bound to a concrete slab with a thin layer of plaster of Paris and (as far as the authors of this paper are aware of) they have not yet published the results of in situ measurements of the dynamic stiffness of pavements.

Taking into account the maximum grain size of aggregate in asphalt mixtures used in typical road surfaces (up to 11–14 mm), the dimensions of the circular plate were considered insufficient for mediating in the transfer of dynamic forces to the tested samples adopted in [15]. Moreover, the stresses on road surfaces loaded with vehicle traffic are much greater (about 2.0 MPa—according to the result of numerical simulations presented in [23])**,** than the loads occurring in the case of materials used for floating floors (0.4–4.0 kPa)—for which the dedicated dynamic stiffness test procedure has been developed [14]. Reliable determination of the dynamic stiffness of bituminous composites requires a larger diameter of the circular plate through which the loads from the exciter are transmitted, and much larger values of both static and dynamic loads that can be generated. That is why the authors of this study developed their own test stand for broadband measurement of dynamic stiffness of vibro-insulating materials and bituminous composites using the Resonant and Non-resonant Methods. The test stand and the results of preliminary tests related to the laboratory testing of the influence of different static and dynamic loads on the dynamic stiffness of cylindrical samples of rubberised asphalt mixtures, stone mastic asphalt (SMA) mixtures and asphalt concrete (AC) were presented in [2]. The obtained results were characterized by very high repeatability and strong coherence of signals recorded with the use of an impedance head. 

## 3. Test Stand for Measuring Dynamic Stiffness

At the stage of modernisation of the test stand for dynamic stiffness determination, an assumption was made about the possibility of carrying out measurements with the Resonant and Non-resonant Methods in laboratory conditions (on samples of any shape and dimension) and with the Non-resonant Method in field conditions implemented directly on various types of road surfaces.

In the test stand, described in detail by the authors in [2], the input excitation in the exciter was transferred to the tested structure (road surface or tested samples) via a drive rod (a so-called stinger) and an impedance head mounted at the opposite end (according to guidelines [24]), which allowed for the simultaneous measurement of driving-point force and acceleration.

The impedance head is not directly attached to the tested structure. It is usually screwed to the dedicated hexagonal mounting bases with a diameter of over a dozen mm, which are bound to the tested structure. Taking into account the typical size of the aggregate grains used in bituminous mixtures and taking into account the operating ranges of the TIRA TV 51144IN inertial exciter (311 N in the case of a generated random signal) as well as the PCB 2888D01 impedance head, it was considered a reasonable compromise to transfer the loads from the exciter to the tested samples or the road surface through a steel circular plate with a diameter of 30 mm, which provides over 4.5 times the load transfer surface (7.07 cm^2^) than that adopted in the test stand described in [15].

In a previous study [2] it was emphasized that to improve the test stand it is necessary to conduct further tests (on samples in laboratory conditions and in situ on road surfaces) with the use of a stiffer stinger.

In further tests, two atypical stingers were used: one made of stainless steel; and the other made of polyamide.

**Figure 2:**
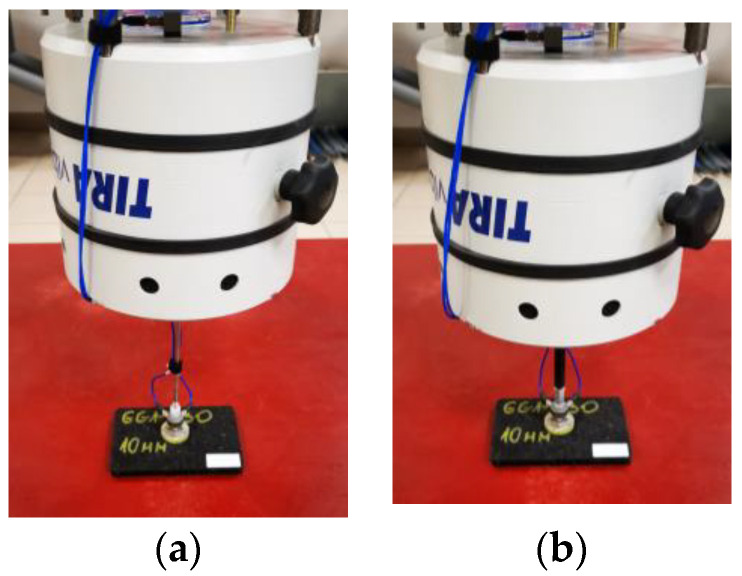
View of the test stand with the stinger mounted: (**a**) made of stainless steel; (**b**) made of polyamide.

Dynamic stiffness tests using the above-mentioned stingers were carried out on SEMAG-ACOUSTIC mats, type GF1-730 and GG1-930 (6 mm and 10 mm thick). The test stand with mounted stainless steel and polyamide stingers is shown in Figure 2.

As a result of the research, it was found that in the case of asphalt mixtures, a much better solution is to use an atypical stinger made of polyamide (with a diameter almost twice as large as the standard stinger made of nylon) or made of stainless steel. Therefore, in further testing, whether carried out on bituminous samples in laboratory conditions or directly on the road surface, a stinger made of rigid polyamide was used.

**Figure 3:**
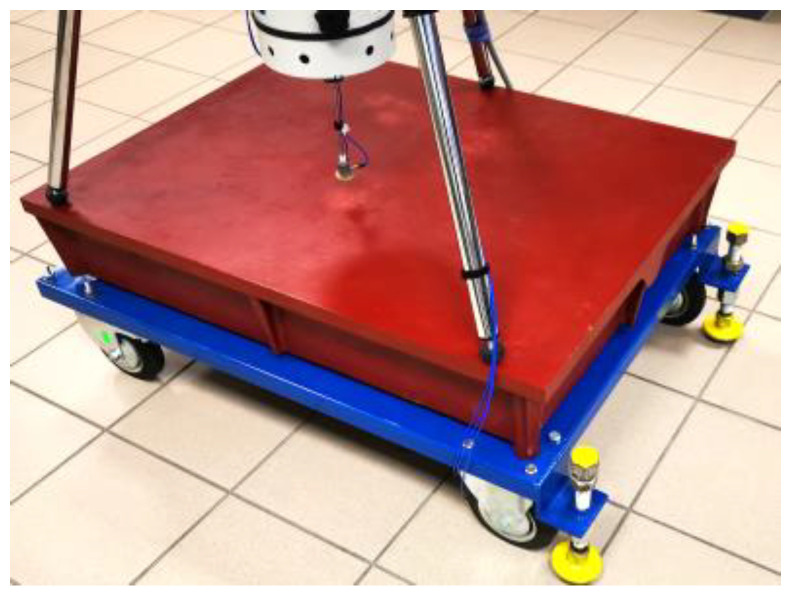
Base of the test stand (100 cm × 75 cm) with a visible supporting structure with a set of 3 swivel wheels and a three-point screw mechanism used in the measurement mode.

In order to increase the mobility of the test stand in laboratory conditions, a massive cast iron measuring/scribing plate (the so-called test stand base) was mounted on a custom designed steel support structure equipped with a set of three swivel wheels, which allows researchers to move the stand freely. Additionally, a three-point mechanism for precise screw lifting (for transport/displacement) and lowering was mounted on the steel supporting structure to secure it in a horizontal position on the laboratory floor for the duration of measurements (in the so-called measurement mode). The supporting structure of the measuring base is shown in Figure 3.

**Figure 4:**
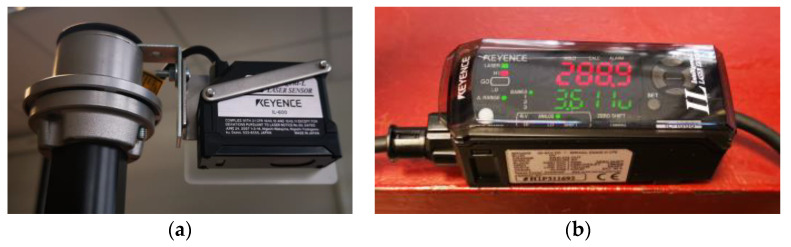
Laser displacement measurement system by Keyence International (Mechelen, Belgium): (**a**) sensor head type IL-600; (**b**) amplifier type IL-1000.

Other modifications were also made to the test stand. Instead of the 32-channel SCADAS Recorder data acquisition system from SIEMENS, a 16-channel SCADAS Mobile measurement system (Plano, TX, USA) was used (with 24-bit measurement cards type VB8-II with a dynamic range of 130 dB and a signal-to-noise ratio of at least 106 dB). Additionally, a control measurement was introduced based on the CMOS Multi-Function Analogue Laser Sensor—IL series produced by Keyence International, with an IL-600 sensor head (measuring range from 200 to 1000 mm) and the IL-1000 type amplifier. The laser displacement measurement system is shown in Figure 4.

To power the data acquisition system and the amplifier and exciter in the field, a Honda EU20i single-phase portable generator with a nominal power of 1.6 kW (max 2.0 kW), IP23 protection rating, and weight of approx. 21 kg was used.

## 4. Research Methodology

The study was divided into two stages. In the first stage (stage No. 1), measurements of dynamic stiffness were carried out directly on selected road surfaces (in situ). In the second stage (stage No. 2), the dynamic stiffness was determined in laboratory conditions on samples taken at the in-situ test sites.

In situ measurements (stage No. 1) were carried out in Poland on the experimental section of the road near Kartoszyno (on 17 March 2021) and on pavements located in the Bialystok University of Technology campus (on 30 April 2021). In the case of the experimental section in Kartoszyno, four test sites were selected on pavements with a poroelastic wearing course made of PSMA5 and PSMA8 with SMA11 and PA11 as binder courses. At the Bialystok University of Technology campus, research was carried out in one location with a typical asphalt concrete (AC) surface on a concrete base course.

**Figure 5:**
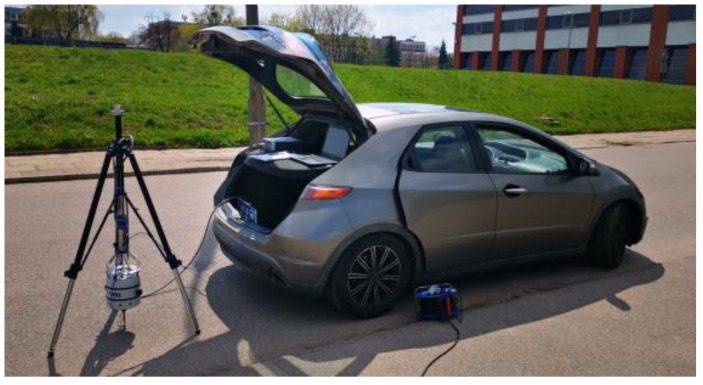
Test site No. 5—asphalt concrete pavement (AC) on a concrete base course (internal road at the Bialystok University of Technology campus).

In each of the adopted test sites, three measurements were taken at points separated by at least 30 cm from one another. At each test site, samples were taken for further testing in laboratory conditions. The mobile test stand and exemplary test sites are shown in Figure 5.

**Figure 6:**
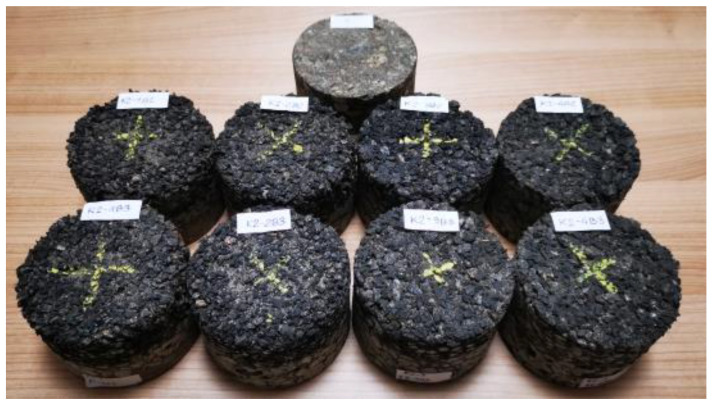
Specimens taken from pavements made to standardized dimensions.

In the second stage of the study (stage No. 2), measurements of dynamic stiffness were carried out in the laboratory of the Bialystok University of Technology on samples taken from road pavements where in situ measurements were carried out (from five test sites). The samples drilled from the pavement were made to the standardized dimensions (diameter: 10 cm; height: 7 cm) (Figure 6). The thickness of the layer of the PSMA5 and PSMA8 poroelastic mixtures was 3 cm on average, and the thickness of the binder course of SMA11 and PA11 was 4 cm on average.

In Table 1 average values of selected parameters characterizing bituminous mixtures are summarised, based on laboratory tests of samples taken from the tested pavements, made at the Bialystok University of Technology [25,26]. Unfortunately, the article does not contain more detailed information on the composition and production technology of the poroelastic mixtures tested due to the fact that such data are kept confidential by the manufacturer of the poroelastic mixtures and the surface contractor.

For each of the samples, three measurements of dynamic stiffness were carried out with the use of identical data acquisition settings and the signal generated by the exciter—as in the case of in situ measurements.

To maximize the possibilities of the test stand, measurements of dynamic stiffness were carried out in a frequency bandwidth that is twice as wide (2048 Hz) as in previous tests [2]. The same number of spectral lines was assumed to obtain the Fast Fourier Transform (FFT) resolution of the recorded signal at the level of 1 Hz—which is quite sufficient considering the bandwidth, acquisition time of a single spectrum (1 s) and the assumed total measurement time including 500 spectra measurements—necessary for later averaging and obtaining results with at least 90% certainty that the random error of the calculated frequency response function will be less than ±5% (according to [24], with the coherence of the recorded signals at the level of 0.8, a minimum of 178 spectra averages are required to be 90% confident that the random error of the computed magnitude of the frequency response function is less than ±5%).

In accordance with the recommendations of ISO 7626-2 [24] the time-domain weighting of the signals was undertaken using the Hanning time-weighting function. The total time of data acquisition was not shortened in the measurements through the enabling the Overlap function.

Analysis of preliminary test results show that the coherence of the recorded signals for both in situ tests and lab samples of the pavement are close to 1.0 (in each case significantly exceeding the value of 0.9). With 500 averages and such a high level of coherence of the recorded signals, the random error should be significantly lower than ±5% [24].

The ISO 7626-2 [24] and PN-EN 29052-1 [14] standards allow for both sinusoidal (at constant amplitude and variable excitation frequency), random (white noise) or shock excitation. In the conducted measurements, a random excitation in the form of white noise was used. The parameters of the generated random signal were set in a slightly narrower band than the recorded vibrations—that is from 2.56 Hz to 2000 Hz. The signal amplification after preliminary tests was assumed at the level of 0.2 V—at which the permissible values of the acceleration of the exciter vibrations and the amplitude of the force limited to +/−100 N are not exceeded.

With the applied static load from the exciter at the level of about 10 kg and the diameter of the load plate of 30 mm, the sample load at the driving-point was 138.8 kPa.

The registration and analysis of the recorded data was carried out using the LMS Test.Lab Spectral Testing software (version 16A, Siemens PLM Software, Plano, TX, USA).

## 5. Field and Laboratory Test Results

**Figure 7:**
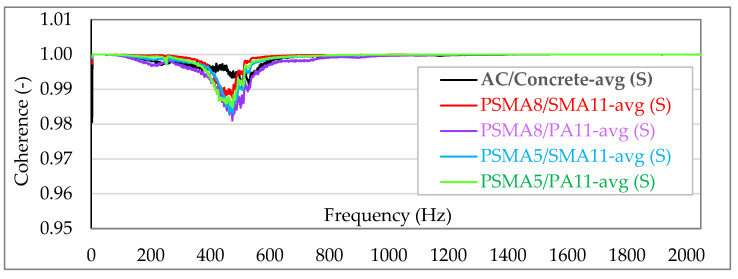
Summary of the coherence for all asphalt mixtures—in situ measurements (S).

**Figure 8:**
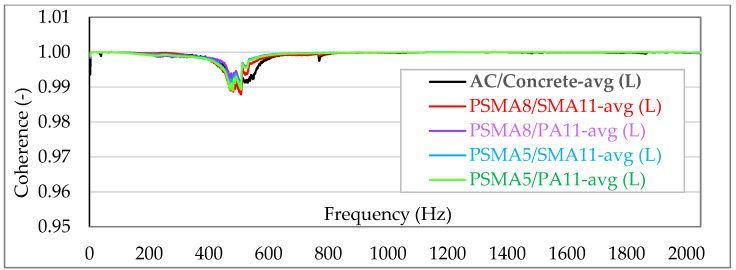
Summary of the coherence for all asphalt mixtures—laboratory measurements (L).

Figure 7 and Figure 8 show the averaged coherence functions (avg) for each of the tested asphalt mixes—independently for laboratory tests (L) and in situ measurements (S). In order to visualize the differences between the individual measurements, the coherence in the charts is shown linearly on a scale from 0.95 to 1.001.

**Figure 9:**
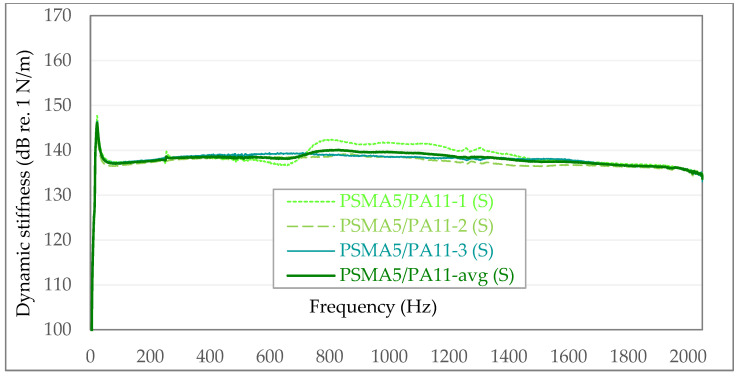
Dynamic stiffness in the case of the PSMA5 poroelastic pavement with PA11 as binder course—in situ measurements (S).

**Figure 10:**
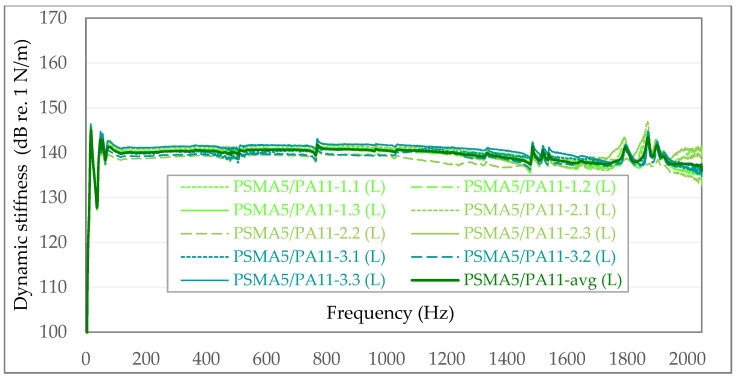
Dynamic stiffness in the case of the PSMA5 poroelastic layer with PA11 as binder course—laboratory measurements (L).

In the following graphs (Figure 9 and Figure 10), the exemplary results of in situ dynamic stiffness measurements (S) and laboratory measurements on samples taken from the above-mentioned sections (L) are presented. The dynamic stiffness in the graphs is presented in [N/m] on the dB/Level scale. To better visualize the differences between individual dynamic stiffness measurements, the graphs are not presented from 0—but from 100 dB re. 1 N/m. In each of the following graphs, the maximum value on the vertical axis was assumed to be 170 dB re. 1 N/m.

**Figure 11:**
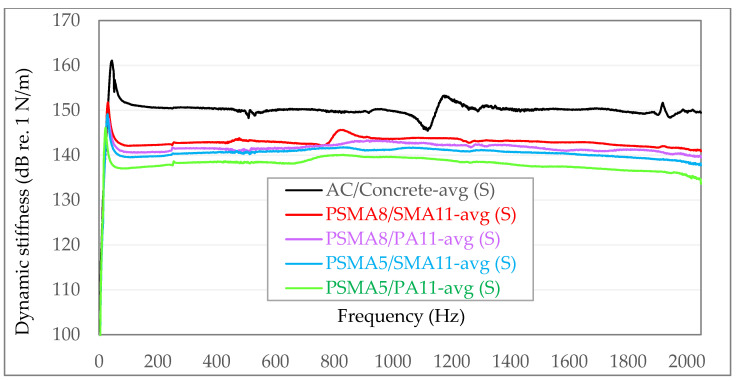
Summary of averaged values of dynamic stiffness from individual measurement series for in situ measurements (S).

**Figure 12:**
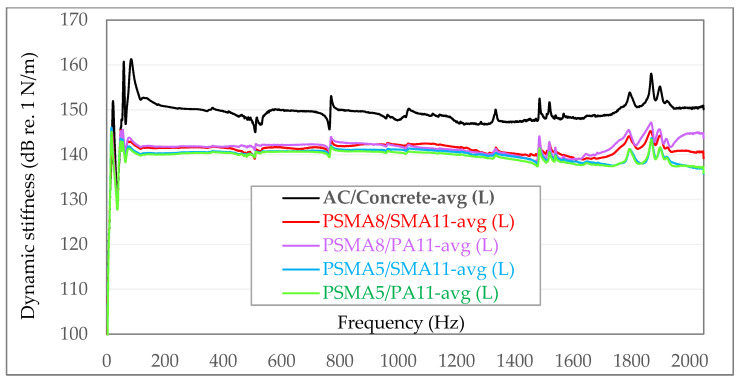
Summary of averaged values of dynamic stiffness from individual measurement series for measurements on samples in the laboratory (L).

To facilitate the visual comparative analysis of the obtained dynamic stiffness, the following diagrams have compiled averaged values from individual measurement series—independently for in situ tests (Figure 11) and laboratory tests (Figure 12).

**Figure 13:**
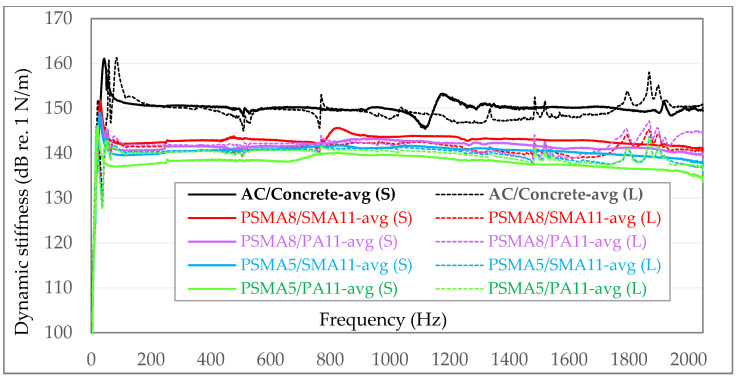
Comparison of the averaged values of dynamic stiffness from individual measurement series for in situ measurements on road surfaces (S) and on samples in the laboratory (L).

To visualize the differences in the dynamic stiffness obtained as a result of field tests carried out directly on asphalt surfaces and tests carried out in laboratory conditions, all averaged values were summarised in one graph (Figure 13):

The dynamic stiffness for selected frequencies (100 Hz, 300 Hz, 500 Hz and 1000 Hz) is also summarised in tabular form (Table 2, Table 3, Table 4, Table 5, Table 6, Table 7, Table 8, Table 9, Table 10 and Table 11). Additionally, arithmetic averages and standard deviations were determined for each of the measurement series.

## 6. Results Analysis

The measurements and analysis of the recorded signals show that in each case there is a very strong coherence between force and acceleration signals (practically in the entire analysed frequency band the values are very close to 1.0). In the most unfavourable case (measurement of the dynamic stiffness on a poroelastic pavement with a wearing course of the PSMA8 with PA11 as binder course), the coherence does not fall below the level of 0.96. According to the guidelines of ISO 7626-2 [24], with the coherence significantly above 0.8 and the use of averages from 500 spectra, a random error of less than ±5% was definitely obtained with 90% confidence level. Coherence at a level so close to 1.0 indicates strong validation of the research results.

Analysing the results of dynamic stiffness measurements carried out directly on the tested poroelastic pavements, it was found that the pavement made of the PSMA5 with the PA11 as binder course was characterized by the lowest stiffness. Dynamic stiffness for selected frequencies in the range of 100–1000 Hz is from 137.1 to 139.6 dB re. 1 N/m. Slightly higher stiffness was obtained in the case of the poroelastic pavement PSMA5 with SMA11 as binder course (139.6–141.3 dB re. 1 N/m). Much higher averaged dynamic stiffness was obtained for the poroelastic pavement made of PSMA8 with PA11 as binder course (140.7–143.0 dB re. 1 N/m). The highest dynamic stiffness in the case of poroelastic pavements was obtained for the PSMA8 with SMA11 as binder course (142.1–143.6 dB re. 1 N/m). A pavement with a wearing course of asphalt concrete (AC) on a concrete base course is characterized by a much higher dynamic stiffness than poroelastic pavements and is in the range of 149.9–151.5 dB re. 1 N/m.

The data summarised in Table 2, Table 3, Table 4, Table 5 and Table 6 show that a satisfactory convergence of the results for in situ measurements carried out within individual measurements series was obtained. The standard deviations of the averaged values of dynamic stiffness did not exceed 1.2 dB re. 1 N/m in most cases.

In the dynamic stiffness tests under laboratory conditions on samples taken from road surfaces, the same trend of changes was found as in the field tests. The lowest dynamic stiffness is characteristic of the PSMA5 with PA11 as binder course (139.9.3–140.5 dB re. 1 N/m). Slightly higher values were obtained for the PSMA5 with SMA11 as binder course (140.0–141.1 dB re. 1 N/m). In the case of PSMA8 with SMA11 and PA11 as binder courses, quite similar levels of average dynamic stiffness were obtained (139.7–142.6 dB re. 1 N/m).

Similarly, as in the case of dynamic stiffness tests of the road surface, also in the case of laboratory tests, the highest values were obtained for samples of asphalt concrete (147.1–155.6 dB re. 1 N/m).

The values of standard deviations for the results of measurements carried out in laboratory conditions on samples of poroelastic layers did not exceed the value of 1.2 dB re. 1 N/m. For asphalt concrete (AC) samples on a cement concrete base course, the standard deviation was 0.6 dB re. 1 N/m for frequencies of 100 and 300 Hz. The significant scatter at higher frequencies (4.5 dB re. 1 N/m at 500 Hz) requires further analysis.

When comparing the averaged dynamic stiffness, it can be stated that there is a good agreement between the values determined in the laboratory tests and the results obtained in situ on road surfaces (Table 12).

There were, however, some differences in the values of dynamic stiffness in the range of certain frequencies. They were caused by different test conditions. In the laboratory, the tests were carried out with the use of a rigid cast iron surface plate, and under in situ conditions, a flexible substructure and subsoil were under the binder course of the pavement.

The determined results confirm the lower stiffness of the PSMA5 (with the maximum grain size of 5 mm) compared to the PSMA8 (with the maximum grain size of 8 mm). In the case of the PSMA5, a more favourable effect of the PA11 as binder course on the dynamic stiffness of the pavement was also noted. In both field and laboratory tests, a significantly higher dynamic stiffness of the asphalt concrete pavement was found.

Based on the findings, it can be concluded that the dynamic stiffness influences the differences in the levels of tyre/road noise between poroelastic surfaces and asphalt concrete surfaces (see Figure 1). Such a statement is acceptable considering the fact that the stiffness of the porous asphalt pavements is closer to that of the SMA and AC surfaces than to the poroelastic pavements, as it was shown in [2]. However, from an acoustical point of view, further research into the stiffness of the mixtures used for the top layers of pavements is necessary.

## 7. Conclusions

As part of the research, a similar trend was found in the distribution of dynamic stiffness of the tested pavements and layers, both in the case of field measurements and tests in laboratory conditions.

The lowest dynamic stiffness is characteristic of the PSMA5 on the porous asphalt PA11 as binder course (the average values for in situ and laboratory tests were 138.3 and 140.3 dB re. 1 N/m, respectively). Slightly higher stiffness was established for the PSMA5 on the SMA11 as binder course (140.5 and 140.6 dB re. 1 N/m). Even higher values of dynamic stiffness were found for the PSMA8 with PA11 and SMA11 as binder courses (141.6 and 142.0 dB re. 1 N/m and 141.5 and 143.0 dB re. 1 N/m). The highest dynamic stiffness in the group of tested mixtures was determined for asphalt concrete (AC) on a cement concrete as base course (150.1 and 150.3 dB re. 1 N/m).

Slight differences in the values of dynamic stiffness in the range of certain frequencies in the in situ and laboratory conditions result from different test conditions. In the laboratory, the tests were carried out with the use of a measuring base (in the form of a rigid cast iron surface plate), and under in situ conditions, a flexible substructure and subsoil were under the binder course of the pavement.

At the same time, satisfactory values of standard deviations within individual measurement series were obtained. Slightly larger dispersions of the values of dynamic stiffness in the vicinity of the mean values were noted in the case of in situ tests.

The findings concerning the dynamic stiffness of poroelastic and asphalt concrete layers made as part of the study confirm its influence on the level of rolling noise. The constructed test stand enables the testing of mineral-asphalt mixtures (including poroelastic mixtures) in laboratory conditions before deciding to use them on a specific road in real field conditions. However, it does not allow for the assessment of the pavement load capacity due to the assumed range of the applied loads. It is necessary to continue the research with the use of the proposed test stand and tire/road noise measurements to develop the relationship between the stiffness and the level of generated sounds.

The conducted measurements and analysis of the results prove the usefulness of the proposed test stand for determining the dynamic stiffness of bituminous mixtures in laboratory and field conditions. This is confirmed by the coherence between the force and acceleration signals at the level of at least 0.96—which indicates a very good validation of the test results with a random error lower than ±5% with 90% confidence level.

## Figures and Tables

**Table 1:** Characteristics of the bituminous mixtures according to research at the Bialystok University of Technology.

Bituminous Mixtures	PSMA5	PSMA8	SMA11	PA11	AC
Coarse aggregate and crumb rubber (>2 mm), % by mass	70.35	68.83	67.79	80.96	56.24
Fine aggregate 0/2, % by mass	10.56	10.43	15.92	6.30	28.92
Filler, fiber and hydrated lime, % by mass	10.88	12.27	9.92	6.02	9.36
Binder soluble, % by mass	8.21	8.47	6.37	6.72	5.48
Air void content, %	28.8	23.6	2.20	15.00	1.80
Bulk density, Mg/m^3^	1.466	1.553	2.453	2.092	2.393
Density, Mg/m^3^	2.059	2.030	2.459	2.358	2.436

**Table 2:** Dynamic stiffness (dB re. 1 N/m) for PSMA5 poroelastic pavement with PA11 as binder course—measurements in situ (S).

Frequency	Sample No.			Average Value	Standard Deviation
[Hz]	1	2	3		
100	137.4	136.6	137.2	137.1	0.4
300	138.5	138.0	138.5	138.3	0.3
500	138.1	138.1	138.9	138.4	0.5
1000	141.7	138.5	138.5	139.6	1.8

**Table 3:** Dynamic stiffness (dB re. 1 N/m) for PSMA5 poroelastic pavement with SMA11 as binder course—measurements in situ (S).

Frequency	Sample No.			Average Value	Standard Deviation
[Hz]	1	2	3		
100	140.0	140.1	138.6	139.6	0.8
300	140.7	140.7	139.7	140.4	0.5
500	140.2	141.1	140.8	140.7	0.5
1000	142.1	140.7	141.0	141.3	0.8

**Table 4:** Dynamic stiffness (dB re. 1 N/m) for PSMA8 poroelastic pavement with PA11 as binder course—measurements in situ (S).

Frequency	Sample No.			Average Value	Standard Deviation
[Hz]	1	2	3		
100	139.6	139.5	143.0	140.7	2.0
300	140.5	141.0	143.2	141.6	1.4
500	140.2	139.0	143.9	141.0	2.6
1000	143.8	141.7	143.6	143.0	1.2

**Table 5:** Dynamic stiffness (dB re. 1 N/m) for PSMA8 poroelastic pavement with SMA11 as binder course—measurements in situ (S).

Frequency	Sample No.			Average Value	Standard Deviation
[Hz]	1	2	3		
100	142.1	142.7	141.5	142.1	0.6
300	142.9	143.2	142.3	142.8	0.5
500	143.5	142.9	143.7	143.4	0.4
1000	143.2	144.8	142.8	143.6	1.1

**Table 6:** Dynamic stiffness (dB re 1 N/m) for asphalt concrete (AC) on a concrete base course—in situ measurements (S).

Frequency	Sample No.			Average Value	Standard Deviation
[Hz]	1	2	3		
100	151.9	152.4	150.2	151.5	1.2
300	150.9	151.9	149.1	150.6	1.4
500	150.1	149.1	148.7	149.3	0.7
1000	148.7	152.5	148.4	149.9	2.3

**Table 7:** Dynamic stiffness (dB re. 1 N/m) for PSMA5 poroelastic layer with PA11 as binder course—laboratory measurements (L).

Frequency	Sample No.			Average Value	Standard Deviation
[Hz]	1	2	3		
100	140.5	140.0	140.3	140.3	0.3
300	140.5	140.2	140.4	140.4	0.2
500	140.1	140.1	139.6	139.9	0.3
1000	140.7	140.3	140.5	140.5	0.2

**Table 8:** Dynamic stiffness (dB re. 1 N/m) for PSMA5 poroelastic layer with SMA11 as binder course—laboratory measurements (L).

Frequency	Sample No.			Average Value	Standard Deviation
[Hz]	1	2	3		
100	139.9	141.1	140.9	140.6	0.6
300	140.0	140.9	140.7	140.5	0.5
500	138.8	141.0	140.3	140.0	1.1
1000	140.9	141.5	141.0	141.1	0.3

**Table 9:** Dynamic stiffness (dB re. 1 N/m) for PSMA8 poroelastic layer with PA11 as binder course—laboratory measurements (L).

Frequency	Sample No.			Average Value	Standard Deviation
[Hz]	1	2	3		
100	143.2	142.1	141.8	142.4	0.7
300	142.7	141.6	141.3	141.9	0.7
500	142.4	140.5	142.3	141.7	1.1
1000	142.9	141.4	141.4	141.9	0.9

**Table 10:** Dynamic stiffness (dB re. 1 N/m) for PSMA8 poroelastic layer with SMA11 as binder course—laboratory measurements (L).

Frequency	Sample No.			Average Value	Standard Deviation
[Hz]	1	2	3		
100	142.3	141.7	141.9	142.0	0.3
300	142.1	141.5	141.3	141.6	0.4
500	141.2	139.5	139.7	140.1	0.9
1000	142.2	141.9	142.6	142.2	0.4

**Table 11:** Dynamic stiffness (dB re 1 N/m) for asphalt concrete (AC) on a concrete base course—laboratory measurements (L).

Frequency	Sample No.			Average Value	Standard Deviation
[Hz]	1	2	3		
100	155.1	155.4	156.2	155.6	0.6
300	149.5	150.1	150.8	150.1	0.6
500	142.1	148.4	150.9	147.1	4.5
1000	146.6	146.0	150.7	147.7	2.5

**Table 12:** The averaged dynamic stiffness from all measurements in situ (S) and laboratory measurements (L), dB re. 1 N/m.

Wearing Course/Binder Course	S	L
PSMA5/PA11	138.3	140.3
PSMA5/SMA11	140.5	140.6
PSMA8/PA11	141.6	142.0
PSMA8/SMA11	143.0	141.5
AC/Concrete (BUT)	150.3	150.1

## Data Availability

The data presented in this study are available on request from the corresponding author.
